# African Swine Fever in the Russian Far East (2019–2020): Spatio-Temporal Analysis and Implications for Wild Ungulates

**DOI:** 10.3389/fvets.2021.723081

**Published:** 2021-08-05

**Authors:** Olga I. Zakharova, Ilya A. Titov, Andrey E. Gogin, Timofey A. Sevskikh, Fedor I. Korennoy, Denis V. Kolbasov, Levon Abrahamyan, Andrey A. Blokhin

**Affiliations:** ^1^Federal Research Center for Virology and Microbiology, Branch in Nizhny Novgorod, Nizhny Novgorod, Russia; ^2^Federal Research Center for Virology and Microbiology, Pokrov, Russia; ^3^Federal Center for Animal Health (FGBI ARRIAH), Vladimir, Russia; ^4^Swine and Poultry Infectious Diseases Research Center (CRIPA) and Groupe de Recherche sur les Maladies Infectieuses en Production Animale (GREMIP), Faculty of Veterinary Medicine, University of Montreal, Saint-Hyacinthe, QC, Canada

**Keywords:** African swine fever virus, cluster analysis, depopulation, Far East of Russia, genotyping, ungulates, wild boar

## Abstract

African swine fever (ASF) is an emerging viral contagious disease affecting domestic pigs (DP) and wild boar (WB). ASF causes significant economic damage to the pig industry worldwide due to nearly 100% mortality and the absence of medical treatments. Since 2019, an intensive spread of ASF has been observed in the Russian Far East region. This spread raises concerns for epidemiologists and ecologists given the potential threat to the WB population, which is an essential member of the region's wild ungulates and provides a notable share of food resources for predatory species. This study aims to determine the genotype of ASF virus circulating in the region, reveal the spatio-temporal patterns of the ASF outbreaks' emergence, and assess the potential reduction of the regional fauna because of expected depopulation of WB. The first historical case of ASF in the study region was caused by an African swine fever virus (ASFV) isolated from DPs and belonging to Genotype 2, CVR1; IGR-2 (TRS +). Sequencing results showed no significant differences among ASFV strains currently circulating in the Russian Federation, Europe, and China. The spatiotemporal analysis with the space-time permutations model demonstrated the presence of six statistically significant clusters of ASF outbreaks with three clusters in DPs and one cluster in WBs. DP outbreaks prevail in the north-west regions of the study area, while northern regions demonstrate a mixture of DP and WB outbreaks. Colocation analysis did not reveal a statistically significant pattern of grouping of one category of outbreaks around the others. The possible damage to the region's fauna was assessed by modeling the total body mass of wild ungulates before and after the wild boars' depopulation, considering a threshold density of WB population of 0.025 head/km^2^, according to the currently in force National Plan on the ASF Eradication in Russia. The results suggest the total mass of ungulates of the entire study region will likely decrease by 8.4% (95% CI: 4.1–13.0%), while it may decrease by 33.6% (19.3–46.1%) in the Primorsky Krai, thereby posing an undeniable threat to the predatory species of the region.

## Introduction

African swine fever (ASF) is among the most dangerous diseases of swine and is known to cause considerable damage to pig production in many countries worldwide (1–5).

The ASF virus (ASFV) belongs to the *Asfarviridae* family and affects both domestic pigs (DP) and wild boars (WB). At present, the genetic characteristics of the ASFV are represented by 23 genotypes associated with geographical locations, characterizing the complexity of the virus epidemiology (3, 6–9). ASF epidemics currently occurring in Europe can be treated as two clusters of outbreaks. One of the clusters is located in Sardinia (Italy), where the disease was registered in 1978 and is represented by ASFV strains belonging to genotype 1 (10). The second cluster is widespread in North-Eastern Europe and the Russian Federation caused by strains of the ASFV belonging to genotype 2 (4). The latter is highly virulent, causing acute disease and resulting in mortality rates of 94.5–100.0% in both WB and DP (11–15).

**Figure 1:**
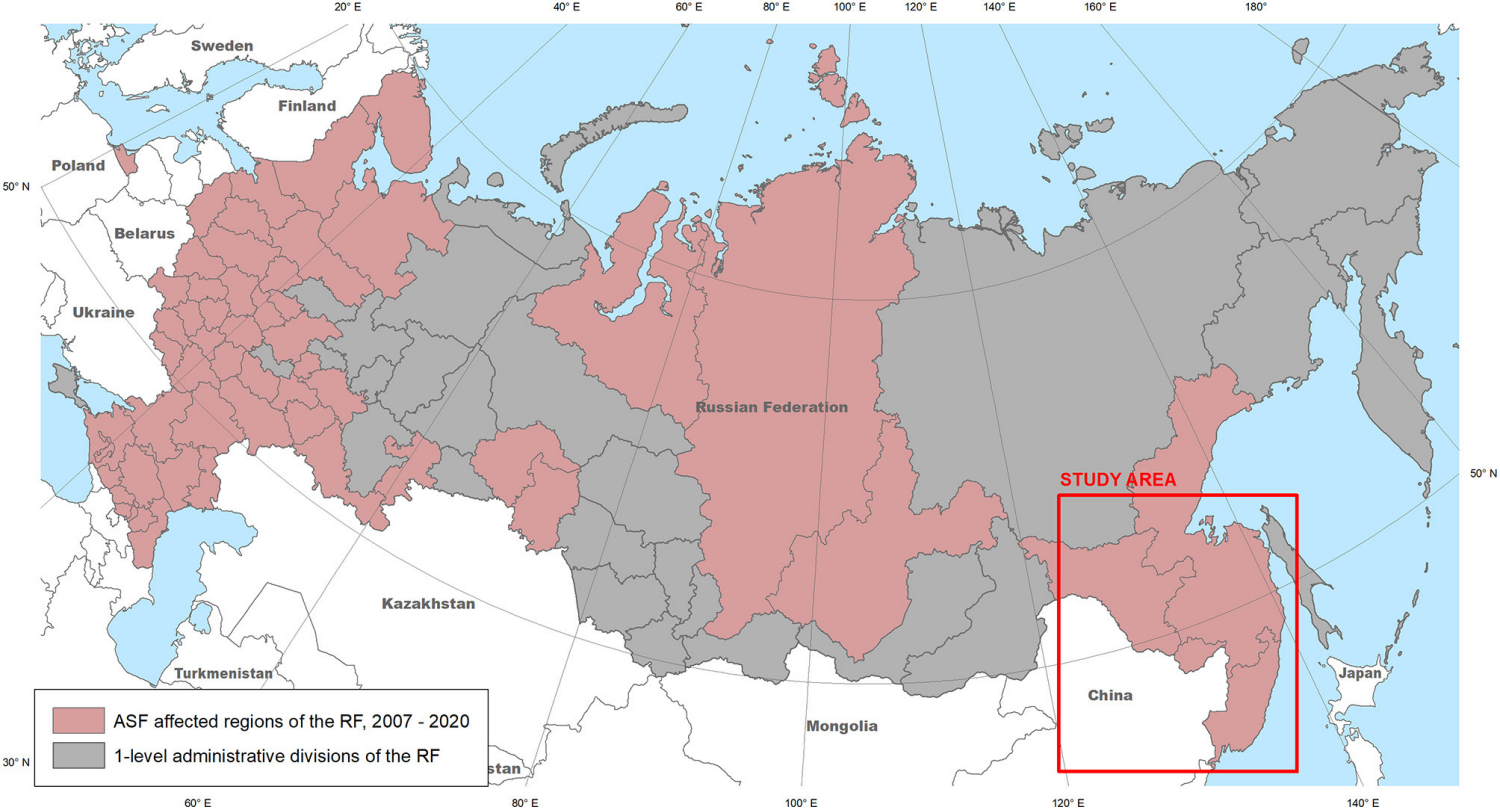
The ASF-affected regions of the Russian Federation, 2007–2020 (source: OIE), and the study area.

The transcontinental transmission of the ASFV in Georgia in 2007, has resulted in the introduction of the disease into the Russian Federation. The first outbreak of the region was registered in WB in the Chechen Republic (November 2007), with subsequent spread of the virus in the Russian Federation and neighboring European countries (16). The nosoareal of the ASF has recently expanded, reaching the Russian Far East (Figure 1) and affecting many countries in South-East Asia (11, 17–21). While insufficient biosecurity on pig farms is considered the main factor in disease spread (22–26), the presence of WB in an ecosystem plays an important role in virus transmission, which is acknowledged by many countries (22, 27, 28).

In some countries with recorded ASF infections, WB populations play the role of an epidemiological reservoir and maintain the virus in the environment. The virus enters WB populations mainly through the animals coming into contact with DP and eating contaminated feed and swill (29, 30).

Wild boars and DP belong to the same species (*Sus scrofa*), as WB are the ancestors of DP. Therefore, the transmission of pathogens is likely bidirectional (31). Wild boars are susceptible to many infections. Although ASF is associated with high mortality, its prevalence in the population does not exceed 3% (32). Nevertheless, every year in the Russian Federation, as in many countries in Europe and Asia, new cases of ASF occur in WB. Proper disposal of infected carcasses (corpses) in compliance with biosafety measures and proper diagnostics for every infected animal are among the essential measures needed to break the ASF transmission cycle (24, 33).

Humans can affect the fluctuations in the numbers of WB, ranging from the maximum possible protection of these animals to their complete eradication. Hunting is the most important risk factor for decline in the WB population, which not only determines their population dynamics, but also has a significant impact on the sex, age, and spatial structure of their populations. Furthermore, WB are the most important hunting trophies and the main prey of large predators (34–37).

One of the measures that has been proposed to control ASF spread is the depopulation of WB, i.e., reducing their numbers to a certain threshold at which the virus' intra-population transmission will stop or significantly slow down owing to the decrease of contact ratio (4, 27, 38). As WB present a significant share of the food resource for many wild predatory species, a decrease in their numbers could disturb the equilibrium of the entire ecosystem and lead to a corresponding decrease in the predator population (39–41). Among the predators of the Russian Far East, the Siberian tiger (*Panthera tigris altaica*) merits mention as a rare protected species, whose population in the region is currently estimated at around 500 individuals (https://wwf.ru/en/regions/amur/amurskiy-tigr/).

Thus, the aims of this study were (1) to conduct an epidemiological and genetic analysis of the ASF situation in the Far Eastern region of Russia, from 2019–2020, and (2) to assess a possible size of WB depopulation and its impact on the whole wild ungulate population of the study region.

## Materials and Methods

### Study Area

**Figure 2:**
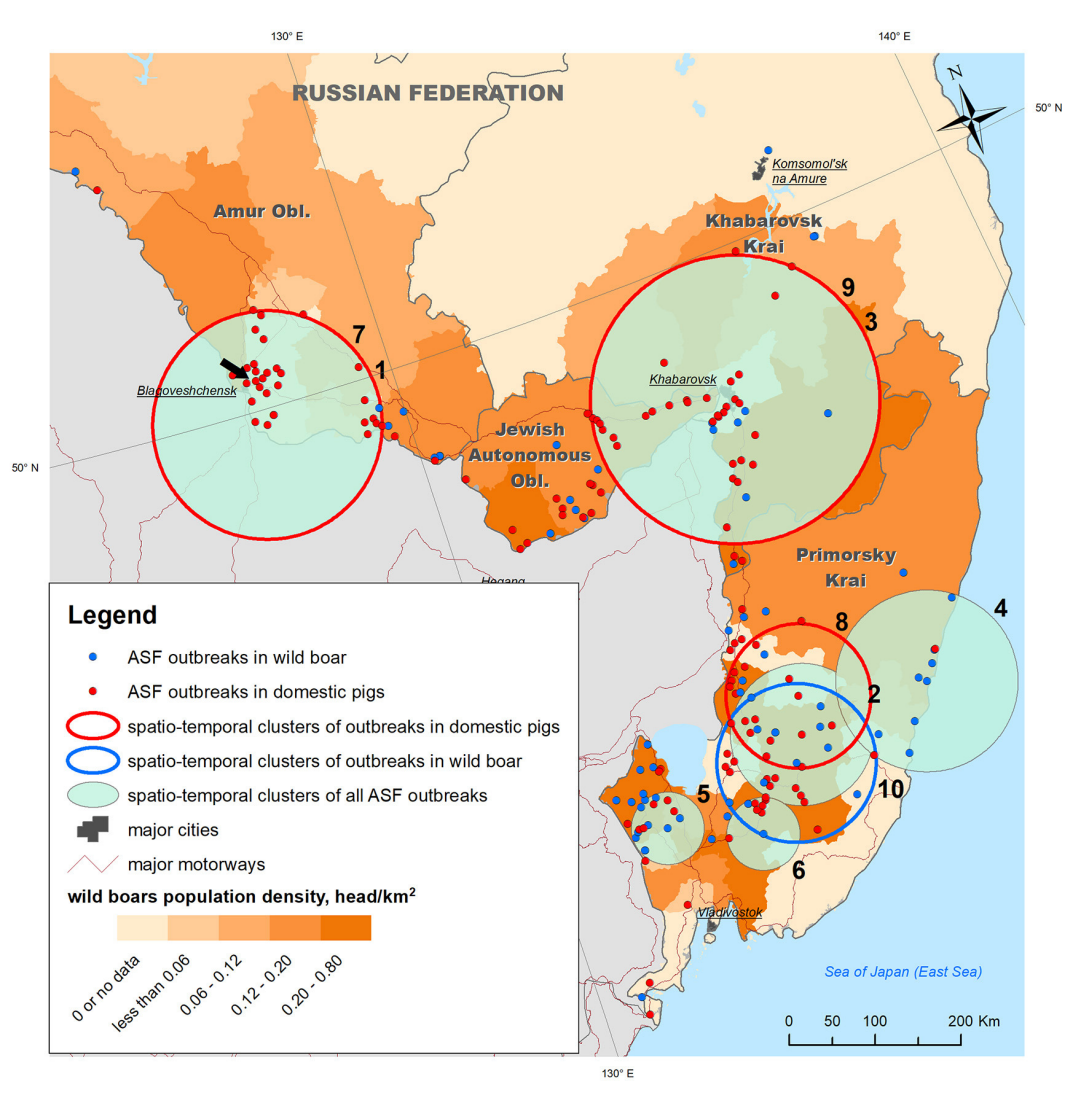
ASF outbreaks (OIE, 2019–2020) and revealed spatio-temporal clusters of outbreaks plotted against wild boar density in the Far East regions of Russia. The black arrow points to the outbreak from which the biological material for ASFV phylogenetic analysis was sampled.

In this study, we considered the ASF epidemic situation in the following four administrative divisions located in the Far Eastern Russian Federation: Primorsky Krai, Khabarovsk Krai, the Jewish Autonomous Oblast, and Amur Oblast (Figure 2). These regions are part of the Far Eastern Federal District of the Russian Federation and were selected because the ASF virus was first detected in this region in 2019. The total area of the study region exceeds 1,350,400 km^2^ with a mean population density of about three people/km^2^. The natural conditions in these areas vary significantly due to the vast extent of the territory from North to South. The climate of the coastal strip in the Far East is relatively warm, humid, and monsoonal. The winters in the Far East are cold and dry, and the summers are hot in continental areas and cool in coastal areas. The climatic conditions in these regions have a huge influence on their economic development. The four aforementioned areas have a rich hydrographic network (http://assoc.khv.gov.ru/regions/information/geography-climate). The leading economic sectors are fishing, forestry, and ore mining. This territory in the Far East contains the country's main ecological reservoir; there are 25 natural reserves and three national parks. A unique feature of this region is the population of Siberian tigers, which is an endangered species listed in the Red Book of the International Union for Conservation of Nature (http://www.nparks.ru/fareast_zapovednik.php).

### Data and Sources

To analyze the epidemic situation, the data on ASF outbreaks notified by the Russian Federation to the OIE from 2019 to 2020 were retrieved from the FAO EMPRES-i database (http://empres-i.fao.org/eipws3g/). The database contains information on 205 ASF outbreaks in the study region for the period from 30 Jul 2019 to 27 Dec 2020, of which 71 (35%) were in WB and 134 (65%) in DP (Figure 2).

Samples of biological material for the phylogenetic study of the ASF virus were obtained from domestic pigs as a result of monitoring studies in an individual private farm in the village of Ust-Ivanovka, Blagoveshchensk District, Amur Oblast and site of the third reported outbreak in the study area (Figure 2).

Data on the population size of WB and other ungulates in the study area were obtained from the Ministry of Natural Resources and Environment of the Russian Federation statistical reports for 2019 (https://www.mnr.gov.ru/en/). Data on wild ungulates' body mass were retrieved from Sokolov (42) and Danilkin (43). The current number of Siberian Tigers in the study area is estimated as ~500 heads, of which 90% live in Primorsky Krai (the World Wildlife Fund (WWF) reports https://wwf.ru/species/amurskiy-tigr/vsye-ob-amurskom-tigre-khozyaine-taygi/). The population density of ungulates was calculated based on the forestry area of the region, which has been retrieved from the Unified Interdepartmental Information and Statistical System UIISS (https://www.fedstat.ru/indicator/38194).

### Sequencing and Phylogenetic Analysis of ASFV

ASF virus “Amur Region/08/19/19,” identified in 2019 during monitoring studies was used in this research. Phylogenetic studies were conducted by the Laboratory for Diagnostics and Monitoring of the Federal Research Center for Virology and Microbiology (https://ficvim.ru/en/). Confirmation of the presence of viral genome fragments in nucleic acid samples was carried out by real-time PCR (44). DNA was extracted using a commercial DNA sorb kit (Interlabservis, Russia) in accordance with the manufacturer's instructions. Then, the samples that were identified as positive by real-time PCR were subjected to sequencing of following fragments: *B646L, E183L, I73R/I329L, B602L, EP402R*, and *MGF 505-9R*. Of these, the *B646L* and *E183L* fragments were chosen as genotype determining genes. The other gene fragments were recommended by the OIE ASF reference laboratory (Madrid, Spain) for molecular characterization of ASF virus strains and isolates (8, 45–49).

PCR was performed using specific primers and Quick-Load® Taq 2X Master Mix (NEB, USA). The PCR products were purified using a MinElute Gel Extraction Kit (Qiagen, Germany). DNA sequencing was performed using an ABI 3130 Genetic Analyzer (Applied Biosystems, USA). The samples were prepared using BigDye Terminator v.3.1 cycle sequencing kit followed by a purification step with BigDye XTerminator™ Purification Kit (both from Applied Biosystem, Foster City, CA, USA) according to the manufacturer's instructions.

BioEdit v7.0.4.1. was used to obtain a consensus sequence (50). The sequences were aligned using ClustalX (51). The sequence data of an ASF virus isolate were subjected to BLAST analysis using the NCBI BLAST tool (https://blast.ncbi.nlm.nih.gov/Blast.cgi) and were compared with sequences of commonly used reference strains and other strains represented in the GenBank (https://www.ncbi.nlm.nih.gov/genbank). Phylogenetic trees were constructed using the maximum likelihood method in which phylogenetic distances were estimated using Kimura's two-parameter model (K2P) (52) in the MEGA 7 program (53).

### Space–Time Data Analysis

A space–time cluster analysis using the space–time permutation model (54) was applied to detect a potential clustering of ASF outbreaks within the study area. This type of analysis allows circular areas to be identified where the observed concentrations of outbreaks over a certain time period are significantly higher than would be expected based on the null hypothesis of the random distribution of outbreaks in space and time. The maximum radius and duration of the clusters were taken to be 50% of the whole study area and period, respectively. Clusters where the observed distribution of outbreaks differed from the hypothetical random distribution at levels of significance >95% (*p* ≤ 0.05) were considered statistically significant. The ODE value (observed/expected) expresses the ratio of the number of outbreaks observed in a cluster to the expected one. Clusters were detected for: (i) all outbreaks; (ii) DP outbreaks only, and (iii) WB outbreaks only.

To visualize the movement of the ASF epidemic since its first emergence within the study area, we applied mapping of the ASF outbreaks' median centers by month using the Median Center GIS (Geographic Information Systems) procedure (55). For each month of the analyzed period, this tool calculates a median point that provides a minimized overall Euclidean distance to all other ASF outbreaks' locations. This median center can be sought as a conventional measure of central tendency less sensitive to spatial outliers than a mean center.

To explore a potential relationship between the ASF outbreaks in DP and WB, we applied a GIS technique named Colocation Analysis. This technique measures local patterns of spatial association between two categories of point features using the colocation quotient statistics (56–58). Given two categories of interest in the study area, namely A and B, the colocation quotient (CQ) expresses a local proportion of category B points within a defined neighborhood of category A points. Category B points are then randomly permuted within the whole study area to estimate whether their observed distribution differs from a random one and to obtain a *p*-value. If the above proportion is higher than a global proportion of category B, the CQ will be >1. Otherwise, the CQ will be below 1. The *p*-value obtained determines the statistical significance of the revealed pattern. Given that colocation analysis is not symmetric, we explored relationships between (i) DP and WB, and (ii) WB and DP outbreaks. To provide a neighborhood for the CQ calculation we tested circular bands of the radius equal to the mean neighboring distance for the whole set of ASF outbreaks (i), and of the radius equal to 150 km (ii) based on the previous studies on ASF outbreaks clustering (59, 60). To add an epidemiological meaning to the analysis of relationship between ASF outbreaks, we analyzed colocation using space-time window accounting for a time span of 2 weeks before and after the analyzed outbreaks that reflects our assumption on the average duration of infectious period in acute ASF course (https://www.oie.int/app/uploads/2021/03/african-swine-fever.pdf). This method reflects an assumption that a certain ASF outbreak might be epidemiologically related to a previous outbreak of the other category within the allowed time period (i.e., an outbreak in DP might be resulted by bringing the virus by infected WB from close neighborhood; or WB outbreak might be caused by contaminated waste or improperly utilized pig carcasses from a preceding DP case).

### Estimation of the Size of the Expected WB Depopulation

According to some studies, decreases in wild boar (WB) populations have been shown to be a requisite measure to prevent the transmission of ASFV in these populations (3, 6, 24, 37). In the currently in force national guidelines to prevent and eradicate ASF in the Russian Federation (61), a value of 0.25 boars/1,000 ha (0.025 boars/km^2^) is recommended as a threshold host density that should be maintained in order to interrupt the circulation of the ASF virus in the WB population within an infected area. Assuming that such a measure may be undertaken in the study region, in this paper we used the above value to estimate the expected size of the WB depopulation.

### Estimation of the Possible Decrease in the Total Mass of the Wild Ungulates

To estimate the possible reduction of the food resource for predatory species of the region, a total mass of ungulates living in the study area was assessed: (1) in 2019, before the ASF virus was introduced to this region; and (2) after the decrease of WB number due to expected WB depopulation, considering a threshold density of 0.025 head/km^2^. Wild boar, musk deer, roe deer, wild reindeer, moose, and red deer are typical ungulates of the region (39, 40, 62). The body mass of each animal was modeled using a uniform distribution based on maximum and minimum estimates taken from the literature (42). Modeling was performed using a Monte Carlo simulation with 10,000 iterations, which allowed the mean and 95% confidence interval of the ungulates' summary body mass to be estimated. The decrease in the total ungulate mass was assessed: (1) for the whole study area and (2) for only Primorsky Krai, which is home to 90% of the entire Siberian tiger population of the study area and has the highest number of WB.

### Software

The cluster analysis was performed using SaTScan software (59). The statistical processing of the data was performed using MS Office Excel application package (Microsoft, Redmond, WA, USA) with @Risk simulation modeling add-on v.6.3 (Palisade Inc., Ithaca, NY, USA). The spatial analysis and results' visualization were carried out using ArcGIS Desktop 10.8.1 and ArcGIS Pro 2.6.3 (Esri, Redlands, CA, USA).

For phylogenetic analysis, BioEdit v7.0.4.1 software (50) was used to obtain a consensus sequence. Nucleotide sequences were aligned with ClustalX (51). To build a phylogenetic tree, the MEGA 7 package (53) was used. To perform a phylogenetic analysis, the analyzed sequences were compared with homological ASFV gene sequences from GenBank database with BLASTN (http://blast.ncbi.nlm.nih.gov/Blast.cgi).

## Results

### Spatio-Temporal Analysis of ASF in the Far East

The analysis of the ASF spread within the study area from its introduction in July 2019 to December 2020, showed that the disease has become widespread over a large territory in Primorsky Krai, the Jewish Autonomous Oblast, Amur Oblast, and Khabarovsk Krai, and mainly concentrated along the Russian–Chinese border. Distribution of outbreaks shows certain spatial heterogeneity across the study area. Outbreaks in WB were mainly observed in Primorsky Krai and Jewish Autonomous Oblast, both of which have the highest density of WB population, while outbreaks in DP were more evenly distributed across the study region, demonstrating a high concentration in Amur Oblast with no WB outbreaks in the same area.

Space–time cluster analysis revealed six statistically significant clusters of ASF outbreaks; three statistically significant clusters of outbreaks in DP, and just one cluster of outbreaks in WB (Table 1 and Figure 2). Two of all ASF outbreaks' clusters demonstrate full spatio-temporal coincidence with clusters in DP (#1 and 7 in Amur oblast, #3 and 9 in Jewish Autonomous Oblast and Khabarovsk Krai) suggesting that those clusters are formed only by the outbreaks in DP. The clustering pattern is more heterogeneous in Primorsky Krai where four spatio-temporal cluster are formed by a mixture of DP and WB outbreaks, with just single clusters in DP and WB, respectively. The latter clusters do not coincide but demonstrate some spatial and temporal overlap. However, moving north-west along the Russian–Chinese border, DP, and WB outbreaks become more spatially separated with the dominance of DP cases.

**Table 1:** Characteristics of spatiotemporal clusters of ASF outbreaks in domestic pigs and wild boar in the Far East of Russia, 2019–2020.

**Cluster #**	**Territorial coverage**	**Cluster radius**, **km**	**Observed number of outbreaks (in population)**	**Observed to expected rate ODE**	**Start date**	**End date**	**Cluster duration, days**	***P*** **-value**
1 and 7	Amur oblast	138	26^*^ (DP)	4.1 for all outbreaks, 2.8 for DP cluster	16.08.2019	26.09.2019	41	<0.001
2	Primorsky Krai	86	17 (DP+WB)	5.8	28.02.2020	30.07.2020	153	<0.001
3 and 9	Jewish Autonomous Oblast and Khabarovsk Krai	175	29^*^ (DP)	3.0 for all outbreaks, 2.5 for DP cluster	31.07.2020	17.09.2020	48	<0.001
4	Primorsky Krai	110	10 (DP+WB)	8.5	16.10.2020	10.12.2020	55	<0.001
5	Primorsky Krai	44	10 (DP+WB)	6.8	06.12.2019	20.02.2020	76	0.002
6	Primorsky Krai	44	10 (DP+WB)	6.2	21.08.2020	15.10.2020	55	0.007
8	Primorsky Krai	88	12^*^ (DP)	5.9	29.05.2020	30.07.2020	62	<0.001
10	Primorsky Krai	96	10 (WB)	5.0	28.02.2020	15.10.2020	230	<0.001

*DP, domestic pigs; WB, wild boars. Cluster numbers correspond to Figure 2*.

* *Several WB outbreaks within the cluster do not belong to the cluster time period*.

**Figure 3:**
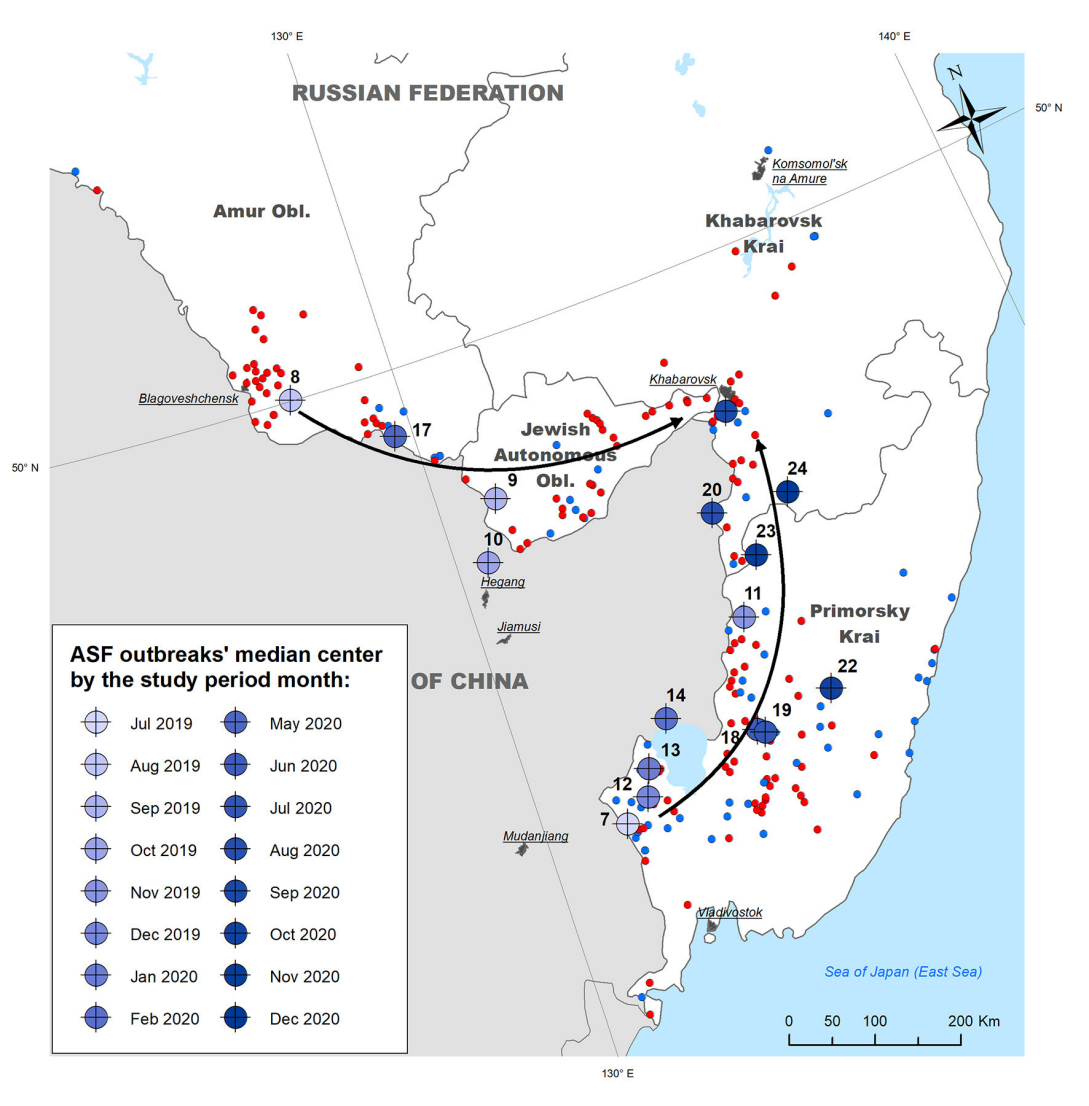
Median centers of ASF outbreaks in the Far East region of Russia by month from July 2019 to December 2020. Arrows indicate the apparent direction of the epidemic movement.

Calculation of the outbreaks' median centers by month demonstrates that ASF almost simultaneously emerged in different regions of the study area adjoining the People's Republic of China: in WB of Primorsky Krai (July 2019) and in DP of Amur Oblast (August 2019). The former region presents a forestry area with the highest density of WB in the region, while the latter is a suburb of Blagoveschensk city that features a direct transport connection with China by a bridge across the Amur River. The direction of median centres' spread suggests consequent epidemic movement from both initial areas north and east with the concentration of most recent outbreaks in Khabarovsk Krai. The direction coincides with the main transportation routes in the region (Figure 3).

**Figure 4:**
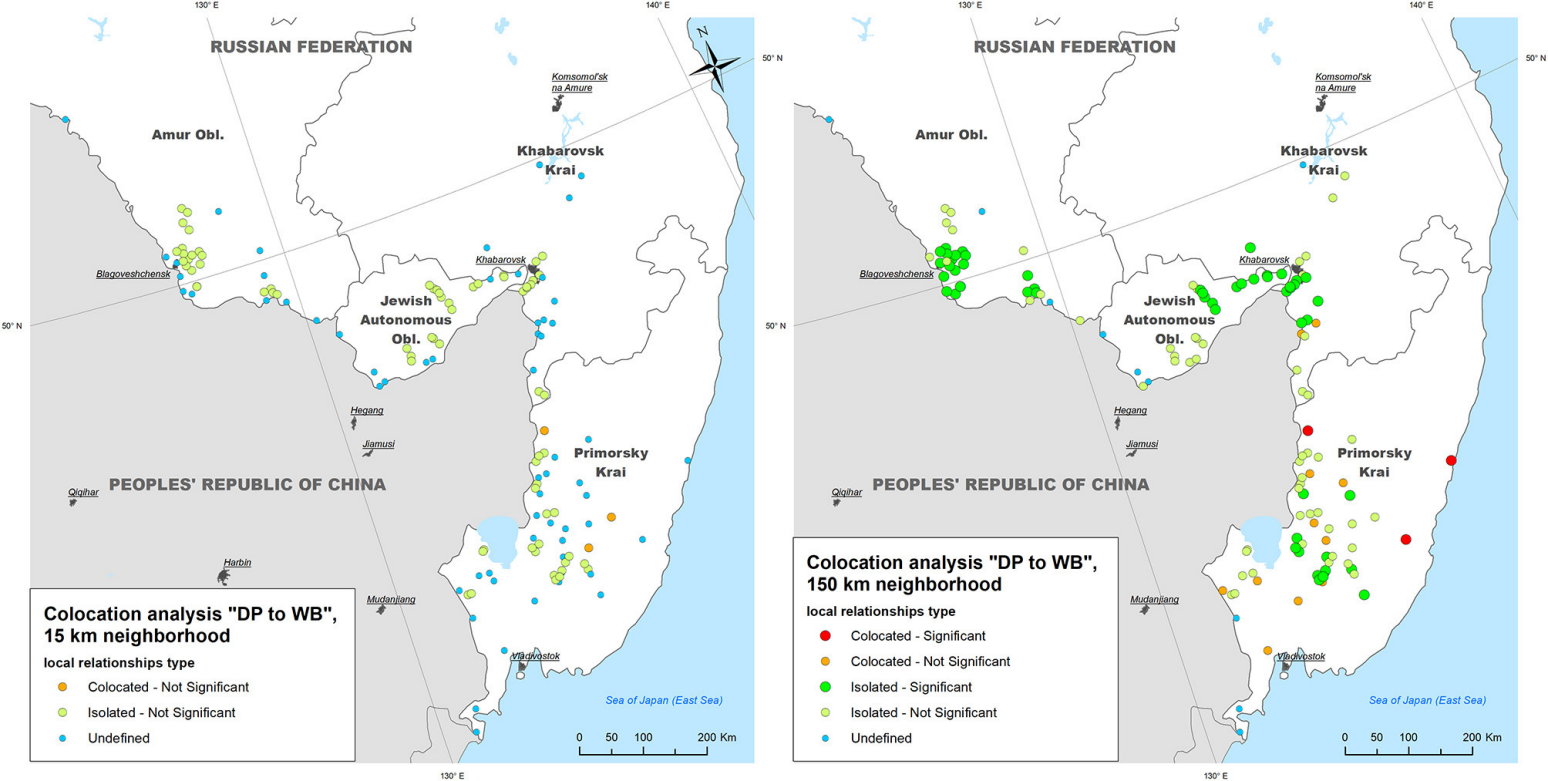
The results of local colocation analysis of ASF outbreaks in domestic pigs (DP) and wild boar (WB). **(Left)** With 15 km as a neighboring distance; **(Right)** with 150 km as neighboring distance.

The mean neighboring distance between all ASF outbreaks within the study area calculated with The Mean Nearest Neighbor GIS tool was found to be 15 km. The colocation analysis of the ASF outbreaks in DP with outbreaks in WB (i.e., testing for an elevated concentration of WB outbreaks in close vicinity to DP outbreaks within 2-weeks time period) showed that:

- Within a neighborhood of 15 km radius, no DP outbreaks were statistically significant collocated with WB outbreaks; only 3 out of 134 (2.2%) DP outbreaks demonstrated statistically non-significant (*p* > 0.05) colocation with WB outbreaks, while 70 (52.2%) DP outbreaks were found to be statistically non-significant isolated;
- Within a neighborhood of 150 km radius, three (2.2%) DP outbreaks were statistically significant (*p* < 0.05) collocated with WB outbreaks (in Primorsky Krai), while 53 DP outbreaks were found to be statistically significant isolated throughout the study area (Figure 4).

**Figure 5:**
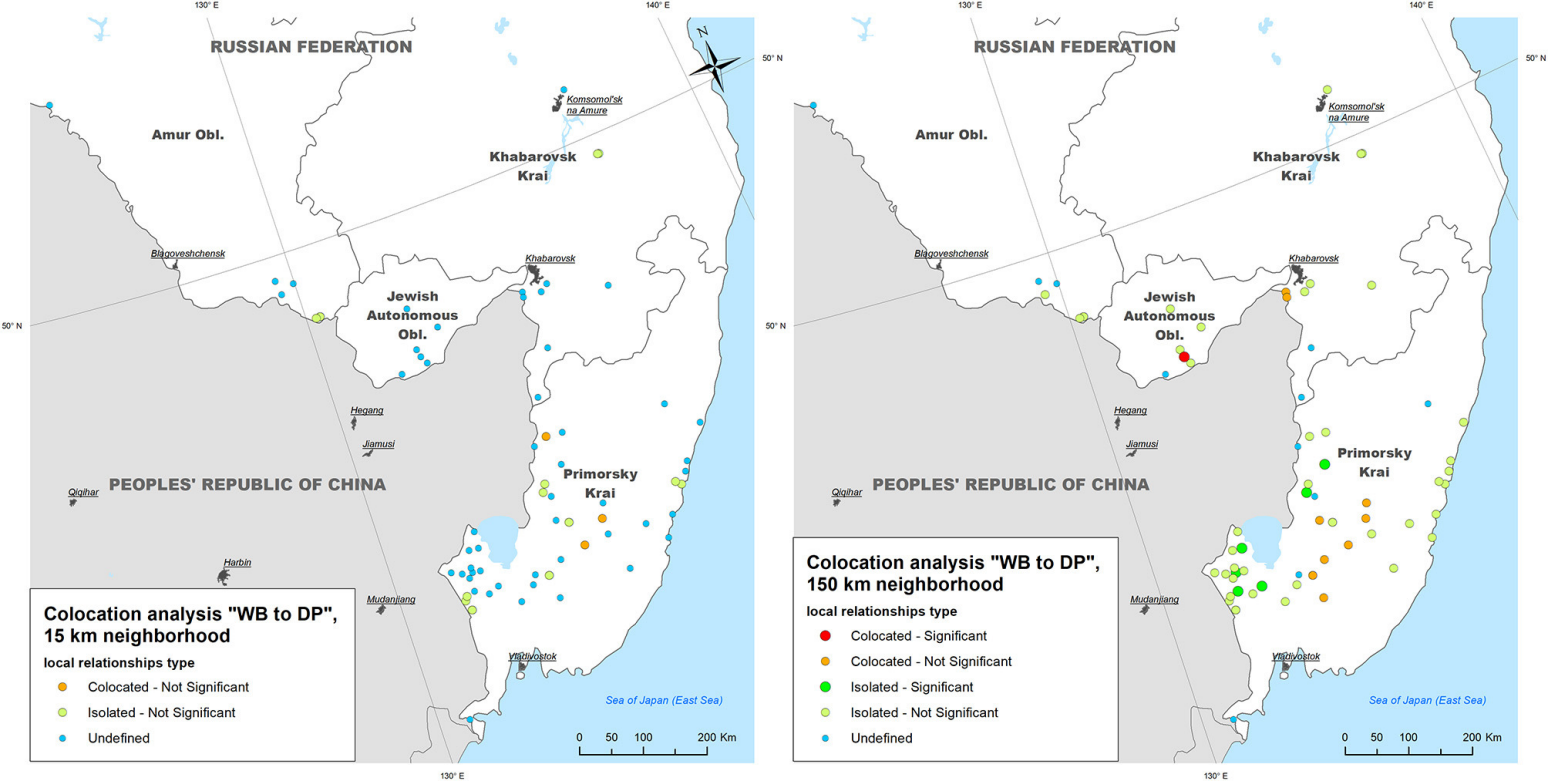
The results of local colocation analysis of ASF outbreaks in wild boar (WB) and domestic pigs (DP). **(Left)** With 15 km as a neighboring distance; **(Right)** with 150 km as neighboring distance.

The colocation analysis of WB outbreaks with DP outbreaks (i.e., testing for an elevated concentration of DP outbreaks in close vicinity to WB outbreaks within 2-weeks time period) demonstrated that:

- Within a neighborhood of 15 km radius, no WB outbreaks were found to be statistically significant collocated with DP outbreaks, while three (4.2%) and 17 (23.9%) of WB outbreaks were statistically non-significant collocated and isolated with DP outbreaks, respectively;
- Within a neighborhood of 150 km radius only one (1.4%) WB outbreak was statistically significant collocated with DP outbreaks (in Jewish Autonomous Oblast), and six (8.5%) WB outbreaks were statistically significant isolated (in Primorsky Krai), while 10 (14.0%) and 43 (60.6%) WB outbreaks were statistically non-significant collocated and isolated, respectively (Figure 5).

### Sequencing and Phylogenetic Analysis for ASFV

The*B646L, E183L, I73R/I329L, B602L, EP402R*, and *MGF 505-9R* gene sequences of the Amur/19.08.19 strain were deposited in GenBank under the accession numbers MT840357, MT840356, MT840354, MT840352, MT840355, and MT840353, respectively.

**Figure 6:**
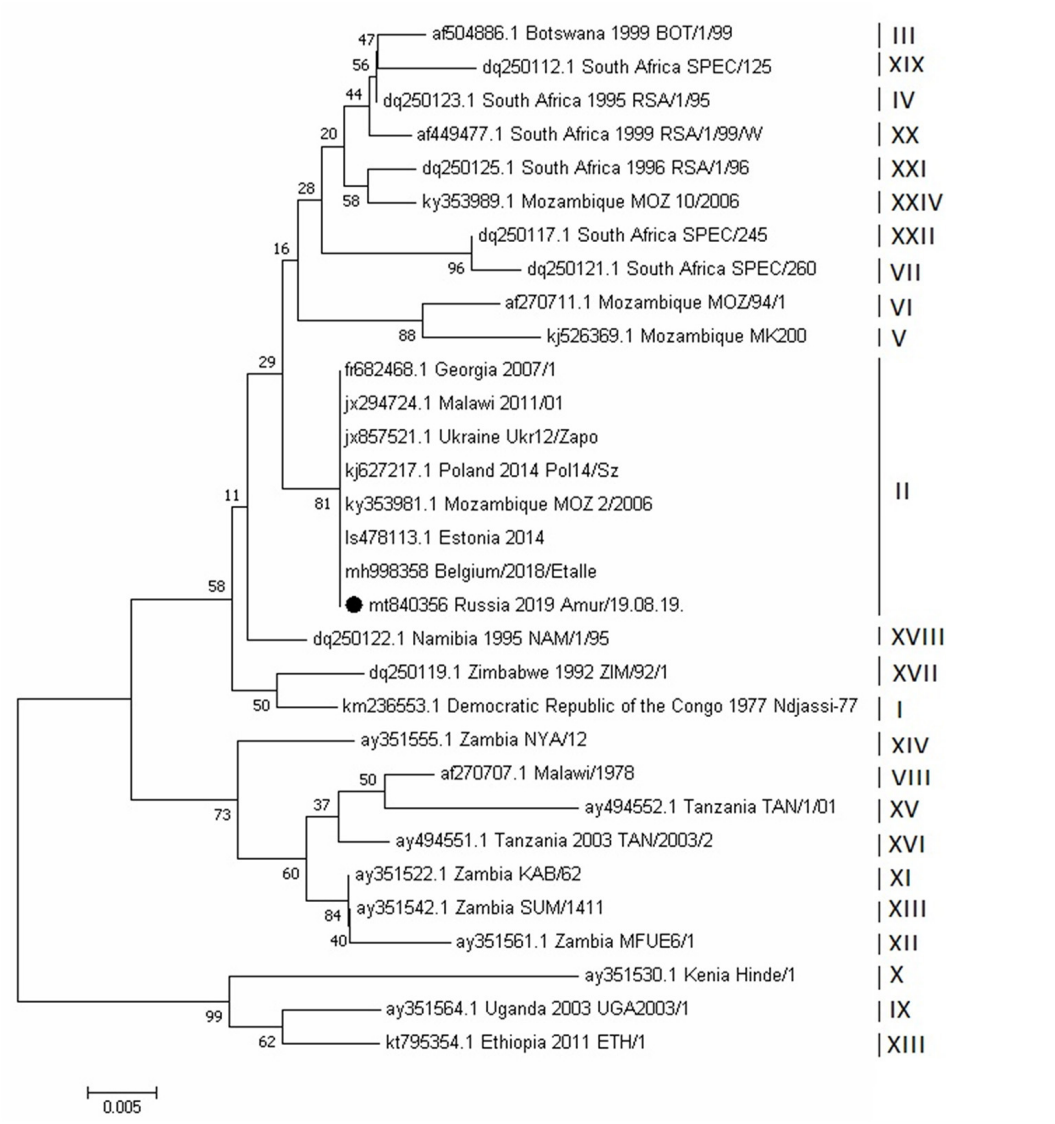
Evolutionary relationships of African swine fever virus strains based on the neighbor-joining phylogeny of the partial p72 gene sequences. The phylogenetic analysis was performed using MEGA7. Bootstrap values (based on 1,000 replicates) for each node are given. GenBank accession numbers, country, and year of collection are indicated for each strain. Corresponding genotypes are labeled I–XXIV. The black circle indicates the African swine fever sequence from Russia, Amur region, 2019. The scale bar indicates nucleotide substitutions per site.

The phylogenetic analysis of ASFV isolates of the Amur/19.08.19 strain was based on a fragment of the *B646L* gene sequence, which encodes the P72 protein. The analysis revealed that this strain belongs to the second genotype. This genotype was initially widely spread in Eastern Europe, when it was first reported in Georgia, in 2007. Genotypic affiliation of the Amur/19.08.19 strain was also confirmed by calculating the phylogenetic tree using the nucleotide sequencing of the *E183L* gene, encoding the P54 protein (Figure 6).

**Figure 7:**
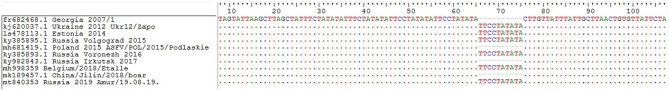
Nucleotide sequence alignment of the partial I73R and I329L intergenic region of the representative African wine fever virus strains. GenBank accession numbers, country, and year of collection are indicated.

No changes were detected in the central variable region (CVR) of the genome, which includes the *B602L* gene. Thus, studied ASFV isolates belong to the CVR1 variant (according to OIE ASF reference laboratory classification [Madrid, Spain]). Moreover, these isolates belonged to the TRS+ (IGR2) variant by classification, based on *I73R/I329L* sequence (Figure 7). Nucleotide substitutions in the sequences of the *EP402R* and *MGF 505-9R* genes were also not detected.

### Assessment of the Decrease in Wild Ungulates' Mass After WB Depopulation

The number of wild ungulates, including the WB, based on the administrative divisions of the study area is shown in Table 2 (as of 2019). The table also presents the estimates of total mass of all ungulates for 2019 as well as after the expected depopulation of WB until a threshold density of 0.025 head/km^2^. The results suggest that the WB population may potentially be reduced by 93% in Primorsky Krai, by 91% in the Jewish Autonomous Oblast, by 46% in Amur Oblast, and by 8% in Khabarovsk Krai. As a result of WB number decline, the total mass of all wild ungulates may decrease by 8.4 % (95% CI: 4.1–13.0%) in the whole study area, while the reduction may be as high as 33.6 % (19.3–46.1%) in Primorsky Krai, which is home to the majority of the Siberian tiger population.

**Table 2:** The results of calculating the mass of wild ungulates based on the reduction in the number of WB in the Far East region of Russia.

	**Musk deer, head**	**Reindeer, head**	**Siberian roe deer, head**	**Moose, head**	**Red deer, head**	**WB (Sus scrofa) by 2019, head**	**Forestry area, km^**2**^**	**WB density before depopulation, head/km^**2**^**	**WB depopulation proportion, %**	**Decrease of total ungulates body mass, % (mean and 95% CI)**
Primorsky Krai	37,588	0	54,046	3,806	33,968	44,517	130,269	0.34	92.7	33.6 (19.3–46.1)
Khabarovsk Krai	38,255	24,268	19,019	61,906	30,730	20,623	755,586	0.03	8.4	0.5 (0.2–0.8)
Jewish Autonomous Oblast	1508	0	14,042	722	3,695	5,973	21,080	0.28	91.2	29.7 (16.2–42.6)
Amur Oblast	24,938	13,794	57,324	22,586	19,201	14,150	305,152	0.05	46.1	3.9 (1.8–6.2)
Body mass minimum, kg	10	80	20	350	93	48				
Body mass maximum, kg	17	180	60	570	148	178				

## Discussion

The observed pattern of the ASF outbreaks' clustering within the study area likely reflects the multiple ways through which the virus may have entered this region. The initial spread started simultaneously in two distant parts of the study region (Primorsky Krai and Amur Oblast) and resulted in a large epidemic involving both DP and WB. The current epidemic situation demonstrates a predominance of DP outbreaks in Amur Oblast, Jewish Autonomous Oblast and Khabarovsk Krai. These outbreaks are mainly concentrated along the Russian—Chinese border and main transportation routes with just several cases of ASF in WB that do not influence the outbreak clustering patterns in these three regions. In contrast, Primorsky Krai shows a mixture of ASF outbreaks both in WB and DP, which are widely spread across its area.

Since the first ASF introduction in the Russian Federation, discussions have been conducted in scientific literature as well as in the media on the leading role of either DP or WB population in the spread of the disease (3, 37, 63). To shed some light on this question using the example of the new epidemic in Far East of Russia, we explored the space-time relationships between DP and WB outbreaks by conducting colocation analysis. This type of analysis looks at the elevated concentration of one category of outbreaks within a close proximity to another and may help to reveal a predominant tendency of DP outbreaks emergence after preceding WB cases, or vice versa. However, our results did not indicate any clear pattern of ASF outbreaks' colocation. The most pronounced results are revealed when taking 150 km as an analysis neighborhood. Most of DP outbreaks, especially in the south-west part of the study area are treated as “isolated” that means an absence or low concentration of WB cases nearby, thus rebutting the hypothesis about the ASF transmission from WB to DP. In Primorsky Krai, the outbreaks' relationships are more heterogeneous, though no obvious pattern is detected as well.

The intensive spread of the ASF virus in the Far Eastern areas of the Russian Federation might have resulted, in part, from insufficient preparedness of the veterinary services and agricultural producers of the region in terms of protection against the virus entry into DP populations. This region has large numbers of poorly protected pig farms, which often practice free range farming techniques, thus with an increased chance for pigs to come into contact with the wild population (64, 65). Therefore, according to the “Cerberus” information system at the Federal Service for Veterinary and Phytosanitary Surveillance, 284 out of 289 registered pig-breeding farms in the Primorsky Krai belong to the first or second biosafety level^1^ suggesting low or no biosecurity; in the Khabarovsk Krai, this number is 32 out of 33 farms; in the Jewish Autonomous Oblast, it is 45 out of 45 farms; and in Amur Oblast, it is 2 out of 2 farms. For comparison, in the European part of the Russian Federation, where measures to control the ASF spread have been in place since 2007, the situation is diametrically opposed. In the Belgorod Region (one of the largest agricultural producers in Russia), only 4 out of 371 farms belong to the first or second biosafety level; in the Nizhny Novgorod Region, this number is 22 out of 118 farms; in the Saratov Region, it is 114 out of 183 farms; and in the Volgograd Region, it is 145 out of 235 farms. Although compartmentalization is currently applied to pig producers on a voluntary basis, the above numbers reflect only a part of all pig farms while still being indicative of the ratio between low- and high-biosecurity holdings in the region.

Although some researchers suggest the threshold density of WB that can stop the ASF spread within the population (4, 27, 66, 67), other authors advocate that achieving this level does not guarantee the cessation of the epidemic chain (24, 37, 68). Currently in force national legislation on the ASF prevention and control supposes the WB density reduction in the ASF affected areas till the threshold of 0.025 head/km^2^, which may result in an intensive depopulation of WB in the study regions. As a side effect of such a depopulation, a decreased availability of food resources for wild predators of the region can be expected. Particularly, it may affect the population of a protected species, Siberian tigers, for which WB present a significant and easily available food source (39, 40, 69, 70). In our study, we aimed to estimate a possible decrease in the wild ungulates' biomass in a case of the reduction of WB numbers. Such approach is based on several assumptions, which are potential limitations of the study. Other possible limitations include the following:

First, the numbers of all the animals were considered at a regional level, with no differentiation according to the various municipal districts (because of a lack of district-level data).
Second, the average weight of each animal was estimated using a uniform distribution (i.e., all values from minimum to maximum were considered to be equally possible), while the real distributions may be quite different.
Third, not all of an animal's mass is edible meat. In reality, no more than 70% of its mass can be considered a food resource (62, 69, 71).
Fourth, we did not account for the age structure of the animal populations, which was partly compensated for by a simulated variation in body mass.
Fifth, according to some studies a prey's population may survive a 1-year withdrawal of 15% to 25% of individual animals with no a long-term impact to its numbers (68). This may indicate that a potential impact of depopulation will be most pronounced in the regions with above 25% estimated depopulation proportion (Table 2).

To carry out a more detailed assessment of the possible impact of a presumable WB depopulation on the equilibrium of the ecosystem, it would be necessary to first have detailed data on the numbers of all animal species at a finer scale (at least the municipal district level), and second, to apply more complex dynamic population models that allow accounting for interactions between the predators and all potential preys.

## Conclusions

Our results demonstrate the emergence of the ASF virus to the Far Eastern areas of the Russian Federation has led to the rapid spread of the disease over a large territory and support a hypothesis of multiple routes of viral introduction into the region. The ASF virus circulating in the study region demonstrates a close homology with corresponding viruses that have been isolated in Europe, Russia, and China. Due to the very dense WB population in this region, the ASF virus can be expected to persist in this population, which may entail the artificial regulation of this WB population to prevent the spread of the disease. The possible reduction of the WB population in Primorsky Krai may lead to a 33.6% (19.3–46.1%) decrease in the total mass of all ungulates, which may pose a threat to the population of predatory species of the region including the protected population of Siberian tigers.

## Data Availability Statement

The genetic datasets presented in this study can be found in online repositories. The names of the repository/repositories and accession number(s) can be found at: NCBI [accession: MT840352-MT840357]. The data on ASF outbreaks were obtained from open sources, which are indicated in the text.

## Ethics Statement

The animal study was reviewed and approved by State Veterinary Service of the Russian Federation. Written informed consent was obtained from the owners for the participation of their animals in this study.

## Author Contributions

DK: conceptualization and project administration. FK, IT, and AB: methodology. FK and OZ: software and data curation, and visualization. FK, AG, and LA: validation. FK and IT: formal analysis. OZ, AB, and TS: investigation. OZ, LA, and FK: writing—original draft preparation. FK, AG, OZ, and LA: writing—review and editing. AG, LA, and DK: supervision. All authors have read and agree to the published version of the manuscript.

## Conflict of Interest

The authors declare that the research was conducted in the absence of any commercial or financial relationships that could be construed as a potential conflict of interest.

## Publisher's Note

All claims expressed in this article are solely those of the authors and do not necessarily represent those of their affiliated organizations, or those of the publisher, the editors and the reviewers. Any product that may be evaluated in this article, or claim that may be made by its manufacturer, is not guaranteed or endorsed by the publisher.

## Footnotes

^1^According to the agricultural producers' biosafety regulations currently in force, all pig-breeding facilities are categorized into four biosafety levels (or compartments). The first and second compartments are considered low or no biosafety holdings, while the third and fourth compartments are large farms with strict biosecurity measures (https://cerberus.vetrf.ru/cerberus/compartment/pub) (Article in Russian).
